# Description of the menstrual cycle status, energy availability, eating behavior and physical performance in a youth female soccer team

**DOI:** 10.1038/s41598-023-37967-4

**Published:** 2023-07-11

**Authors:** Ana Carolina Paludo, Marta Gimunová, Marcos Michaelides, Magdalena Kobus, Koulla Parpa

**Affiliations:** 1 Incubator of Kinanthropology Research, Faculty of Sports Studies, Masaryk University, Kamenice 753/5, Bohunice, Brno, Czech Republic; 2 Department of Physical Activities and Health Science, Faculty of Sports Studies, Masaryk University, Brno, Czech Republic; 3 Faculty of Sports and Exercise Science, UCLan University of Cyprus, Pyla, Cyprus; 4 Department of Anthropology, Faculty of Biology and Environmental Protection, University of Lodz, 90-237 Lodz, Poland; 5 Institute of Biological Sciences, Faculty of Biology and Environmental Sciences, Cardinal Stefan Wyszyński University in Warsaw, 01-938 Warsaw, Poland

**Keywords:** Human behaviour, Quality of life

## Abstract

The aim of the study was to describe the menstrual status and perception, risk of low energy availability (LEA) and the presence of orthorexia nervosa (ON) in youth female from a soccer team. Also, verify the possible effect of LEA and ON on physical performance. Data from 19 female players (14.6 ± 1.42 yr) belonging to a soccer team from Cyprus was taken during pre-season. The menstrual cycle status was evaluated by specific questions, LEA by the Low Energy Availability in Females Questionnaire (LEAF-Q), ON by the ORTO-R questionnaire and physical performance by jump, handgrip and cardiorespiratory exercise tests. Players were separated into the risk of LEA and ON. Comparison and correlation tests were performed with a significance set at p < 0.05. As the main results, 66.7% of players perceived that the menstrual period affects their performance in the game, 83.3% did not communicate with coaches about their menstrual cycle; the prevalence of risk of LEA was 26.3%; players with risk of LEA also presented higher scores ON; neither LEA nor ON presented a significant association with players performance. The findings highlighted that youth players perceived an impact of the menstrual period on performance, but did not communicate with the coach about it. Players with the risk of LEA and high values of ON seem not to be associated with a decrease in physical performance during the pre-season evaluation. Attention is required as the players were assessed once. Monitoring these parameters throughout the sports season is recommended to obtain better clarification about the topic.

## Introduction

Youth female athletes have been considered a population more sensitive to present health issues compared to their counterparts and could be caused by inadequate energy consumption and eating behavior^1^. The presence of low energy availability (LEA) in female youth athletes can result in a delay of puberty, menstrual irregularities, development of harmful eating behaviors^2^, loss of muscle mass, and impairment of physical capacity^3^. Risk of LEA can be found in those athletes who restrict their energy intake, expending a high amount of energy during exercise and/or limiting their food choices^4^. Pathologic eating behavior, as recently named orthorexia nervosa^5^, could be associated with the risk of LEA. Orthorexia nervosa can be interpreted as a pathological fixation on healthy nutrition. The concept was introduced in the 90s but yet not recognized as an eating disorder by some organizations^6^.

To address the aforementioned health issues, the International Olympic Committee attributes the concept of Relative Energy Deficiency (RED-S)^7^, caused by chronically poor energy availability and nutrition^8^. Similarly, the female triad adds up the LEA, menstrual dysfunction and low bone mineral density^7^. Despite the literature describing that aesthetic and weight-dependent sports are more likely to present the components involved in the RED-S and female triad, few studies also reported the presence of menstrual irregularities, negative eating attitudes and the LEA in professional female soccer players^9–12^. Soccer is by far one of the most popular sports played^13^; however, regardless of the increased number of participants in the modality, more information is needed about health issues, especially among female youth.

The description of menstrual cycle status, energy availability and eating behavior still needs to be more outlined in the youth players, as well as the interrelation of these variables on physical performance. Therefore, the aim of this study was to describe the menstrual status and perception, the risk of LEA and the presence of orthorexia nervosa in youth female players from a Cypriot soccer team. Additionally, the comparison and correlation of low energy availability and orthorexia nervosa on physical performance were tested, respectively. We hypothesized an interrelationship among negative menstrual status, low energy availability and poor eating behavior may affect negatively physical performance. Therefore, those players with a risk of LEA will present menstrual irregularities, higher scores of orthorexia nervosa and a significant reduction in physical performance compared to those with no risk.

## Methods

### Participants

Nineteen youth female soccer players belonging to the same team volunteered to participate in the study (mean ± sd: age,14.6 ± 1.42 years; height, 145 ± 50.9 cm; body mass, 54.3 ± 7.64 kg). The team was enrolled in the U-18 Amazon Championship based in Cyprus during the period investigated. Players were not taking prescribed medications or any special dietary supplement and did not report any cardiovascular disease. Parents or legal guardians provided written informed consent after receiving verbal and written information about the study’s procedures, risks and benefits. The study was performed in accordance with the Declaration of Helsinki about humans and was approved by the Cyprus National Committee on Bioethics (CNCB, 7 July 2021). Lastly, all players had medical clearance to participate in training and testing.

### Experimental design

The study presents a cross-sectional design, in which the players were assessed in laboratory conditions in one session, during the pre-season 2022. The first measurement was a survey about descriptive characteristics of training and menstrual cycle status, followed by the energy availability questionnaire (LEAF-Q) and the orthorexia questionnaire (ORTO-R-GR) (Supplemental Material Table 1). One member of the research team supported with questions comprehension, when necessary. After answering the survey, players underwent to anthropometrics measurements and physical tests such as jumps (squat and countermovement), handgrip (right and left hand) and cardiorespiratory tests. Before the measurements, players were instructed to refrain from caffeine and alcohol in the previous 24 h.

### Demographics and anthropometrics characteristics

To characterize the participants, players answered questions about soccer training characteristics (e.g., age of specialization, training time and hours of training), through open and multiple-choice questions. Anthropometric characteristics included body height and body mass. Stature and body mass were measured according to standard procedures^14^ using a wall stadiometer (Leicester; Tanita, Tokyo, Japan) and a standard electronic scale and recorded to the nearest 0.1 kg and 0.1 cm, respectively.

### Menstrual cycle characteristics, perceived effect and communication

Open and close-ended questions were asked to collect players’ information about menstrual characteristics, perceived effect and communication. Players’ menstrual characteristics involved questions about the age of menarche, regular menstrual cycle (natural), use of oral contraception, use of medicine and symptoms during the bleeding period. Furthermore, it was asked if the menstrual cycle affects players’ performance during the training sessions and the matches; and about communication about the menstrual cycle with their coaches and teammates.

### Energy availability

The Low Energy Availability in Females Questionnaire (LEAF-Q) was used to assess the energy availability in the players. The LEAF-Q is a 25-item questionnaire to screen physiological symptoms associated with female athlete triad (Triad) and relative energy deficiency^15^. The questionnaire considers three domains: gastrointestinal function, injuries and menstrual function. A score ≥ 8 indicates a risk of low energy availability and female Triad. The LEAF-Q is validated in female athletes (aged 18–39), involved in ≥ 5 times/week training, reporting an adequate sensitivity (78%) and specificity (90%)^15^. Players answered the English version of the LEAF-Q questionnaire, and any doubt about the translation was supported by a member of the research team that speaks Greek and English.

### Orthorexia nervosa (ON)

The risk of ON was assessed by a Greek version of the ORTO-R questionnaire (ORTO-R-GR)^16^. The ORTO-R is a short version of the ORT-15, a questionnaire developed to identify tendencies of orthorexia nervosa^17^ and consists of 6 items. For each, a Likert five-point scale, with questions receiving between 1 and 5 points is present for possible answers (never, rarely, sometimes, very often and always). Maximal score possible to achieve is 30 points. In this short version, a cut-off point is not suggested for the diagnosis; instead, total scoring is used to indicate more or fewer ON tendencies^18^.

### Physical tests

*Handgrip strength test* A handgrip dynamometer (Takei Scientific Instruments Co., Ltd., Tokyo, Japan) was used to assess the maximum isometric strength of the forearm and hand muscles. The procedure was conducted according to the methods described by previous investigators^19^. Two attempts were performed, and the highest value was retained. *Countermovement Jump (CMJ) and Squat Jump (SJ)* Explosive strength power was assessed with CMJ and SJ tests. The vertical jump performance (CMJ, SJ) was evaluated using OptojumpTM photoelectric cells (Microgate, Bolzano, Italy) based on previously described methods^20^. Each player performed three CMJs and three SJs with the same break between jumps. They were instructed to stand between Optojump bars, and the first task was to perform an SJ with their knee joint bent approximately 100 degrees. They had to descend into a semi-squat position and hold that position for approximately 3 s before taking off. The task was repeated 3 times at 15-s intervals, and the maximum of the 3 trials was recorded for statistical analysis. Each player performed 3 consecutive CMJs with the same break between jumps. In the CMJ, the athlete started from a standing position and initiated a downward movement, followed by an upward movement leading to take off. In both cases, the participant's hands were placed on their waist and swinging of the arms was not allowed. The highest of the three valid jumps was included in the data analysis. *Cardiopulmonary exercise testing* The players completed an incremental maximal exercise testing until they reached exhaustion on a treadmill (h/p/Cosmos Quasar med, H-P-Cosmos Sports and Medical GmbH, Nussdorf-Traunstein, Germany). The players were tested utilizing the modified Heck incremental maximal protocol described by previous investigators^21,22^. A breath-by-breath analysis was performed on the Cosmed Quark CPET (Rome, Italy) system while laboratory conditions were kept constant (temperature 22 ± 1 °C and relative humidity at 50%). The test was terminated once the participant reached volitional fatigue or when there was no variation among the maximal oxygen consumption (VO_2max_) levels while the workload increased. The VO_2max_ was detected following filtering the results to identify the highest value for an average of 10 s. The ventilatory (VT) and respiratory compensation points (RC) were determined using different criteria. The ventilatory threshold was determined through the V-Slope method and was verified at the nadir of the VE/${\dot{\text{V}}}$O_2_ curve. The respiratory compensation point was determined at the nadir of the VE/${\dot{\text{V}}}$CO_2_ curve.

### Statistical analysis

The outcomes were presented using descriptive statistics. For continuing variables, data were presented in mean and standard deviation, as a central tendency and dispersion measure. For categorical variables (e.g., a yes/no answer), data were displayed in absolute and relative frequencies. Pearson-product moment correlation coefficients were used to determine the relationship between ON and physical performance. Comparison between groups of LEAF-Q scores (< 8 points and > 8 points groups) was performed by the independent sample t-test. Cohen's d was calculated to determine the effect size (ES) to present the magnitude of the reported effects of low energy availability risk. ES was interpreted as small (0.2–0.4), medium (0.5–0.7) and large (0.8–1.4)^23^. In addition, it was assumed that when CL does not cross the “0”, a clear difference would be presented. CL crossing the “0” were classified as representing unclear differences^24^. All data were analyzed using the statistic software JAMOVI (2.2.5 version) and significance was set at *p* < 0.05.

## Results

The descriptive players’ attribute is displayed in Table 1. Regarding the training hours, 63.2% of the players train less than 8 h per week (n = 12), and 36.8% train between 8 and 12 h (n = 7).

**Table 1:** Descriptive characteristics of youth soccer players (n = 19).

	Age (year)	Body height (cm)	Body mass (kg)	Body fat (%)	Age of sport specialization (year)	Time training soccer (years)
Mean	14.6	161	54.3	22.1	9.74	4.9
SD	1.42	5.52	7.64	4.69	3.0	2.33

### Menstrual cycle characteristics, perceived effect and communication

Table 2 presents the menstrual cycle characteristics. One player was excluded due to the fact of never being menstruated (primary amenorrhea). It was possible to notice that most of the players have menarche around 12 and 14 years old (83.3%) and it comes naturally (88.9%). Also, most of the players preferred to talk about their menstrual status with teammates (77.8%) but not with the coach (83.3%); no players reported using oral contraception, according to LEAF-Q information (3.1.A question). The team staff consisted of one female (29 years old) head coach and one female strength and conditioning coach and assistant (35 years old) at the time of data collection.

**Table 2:** Menstrual cycle characteristics (n = 18).

Age of menarche		Regular cycle (natural)	
11 years or less	16.7% (n = 3)	Yes	88.9% (n = 16)
12–14 years	83.3% (n = 15)	No/don’t remember	11.1% (n = 2)
MC and training performance		MC and game performance	
Yes	50% (n = 9)	Yes	66.7% (n = 12)
No	50% (n = 9)	No	33.3% (n = 6)
MC and coach communication		MC and teammate communication	
Yes	16.7% (n = 3)	Yes	77.8% (n = 14)
No	83.3% (n = 15)	No	22.2% (n = 4)

*MC* menstrual cycle.

### Energy availability and effect on body fat, ON and physical performance

After the calculation of LEAF-Q scores, the players were separated into two groups, those with scores less than 8 and those with scores equal to and more than 8 points. Five players demonstrated to be at risk of low energy availability according to the LEAF-Q questionnaire. Table 3 described the body mass, ON status (by ORTO-R scores) and physical performance separated by groups. The groups presented a normal distribution for all variables (p > 0.05), thus the data were displayed in mean and standard deviation. A comparison between groups demonstrated that players with a risk of low energy availability presented significantly higher body fat and higher values on the ORTO-R questionnaire compared to their counterparts. The ES for body fat and ORTO-R scores were classified as large (> 1) and clear. No significant differences were found for physical performance (p > 0.05). However, a medium and unclear effect was observed for jumps (SJ and CMJ), and a large and unclear effect was observed for running time and VO_2max_.

**Table 3:** Body fat, ORTO-R scores and physical performance in soccer players divided by LEAF-Q scores (n = 19).

	< 8 points (n = 14)	≥ 8 points (n = 5)	p-value	ES (d cohen)
Body fat	20.7 ± 3.46	26.1 ± 6.74	0.041*	− 1.21 (− 2.24/− 0.07)
ORTO-R (score)	16.3 ± 3.56	20.6 ± 2.97	0.027*	− 1.25 (− 2.29/− 0.11)
SJ (cm)	23.9 ± 4.42	20.6 ± 4.87	0.180	0.73 (− 0.35/1.74)
CMJ (cm)	25.8 ± 4.39	22.4 ± 5.91	0.191	0.71 (− 0.37/1.72)
R-handgrip (kg)	24.3 ± 3.04	25.0 ± 3.35	0.641	− 0.22 (− 1.24/0.81)
L-handgrip (kg)	24.2 ± 3.45	23.9 ± 4.16	0.893	0.11 (− 0.92/1.13)
RT (min)	9.99 ± 1.91	8.27 ± 1.81	0.101	0.91 (− 0.19/1.93)
VO_2max_ (ml/kg/min)	48.2 ± 4.06	44.2 ± 5.43	0.108	0.90 (− 0.19/1.92)

*SJ* squat jump, *CMJ* counter movement jump, *R-handgrip* right handgrip, *L-handgrip* left handgrip, *RT* running time, *VO*_*2*_*max* maximal oxygen uptake, * p<0.05.

### Association between training hours and LEAF-Q scores

The categorical association between training hours and the LEAF-Q status is displayed in Table 4. No significant association was found (p = 0.109). The major percentage (44%) was in players that trained less than 8 h per week which also presented a low risk of low energy availability.

**Table 4:** Association between training hours and LEAF-Q scores (n = 18).

Training hours	LEAF-Q scores		
	< 8 points	≥ 8points	p-value
< 8 h	44.4% (n = 8)	22.2% (n = 4)	0.109
8–12 h	33.3% (n = 6)	0% (n = 0)	

### Correlation between ON and physical performance

As reported in Table 5, no significant correlation was found between the ON and physical performance.

**Table 5:** Correlation between ON and physical performance (n = 19).

ON	SJ (cm)	CMJ (cm)	R-handgrip (kg)	L-handgrip (kg)	RT (min)	VO_2max_ (ml/kg/min)
r	− 0.127	− 0.018	0.174	0.204	0.077	− 0.108
p-value	0.605	0.941	0.476	0.402	0.760	0.670

*ON* orthorexia nervosa, *r* correlation value, *SJ* squat jump, *CMJ* counter movement jump, *R-handgrip* right handgrip, *L-handgrip* left handgrip, *RT* running time, *VO*_*2*_*max* maximal oxygen uptake.

## Discussion

**Figure 1:**
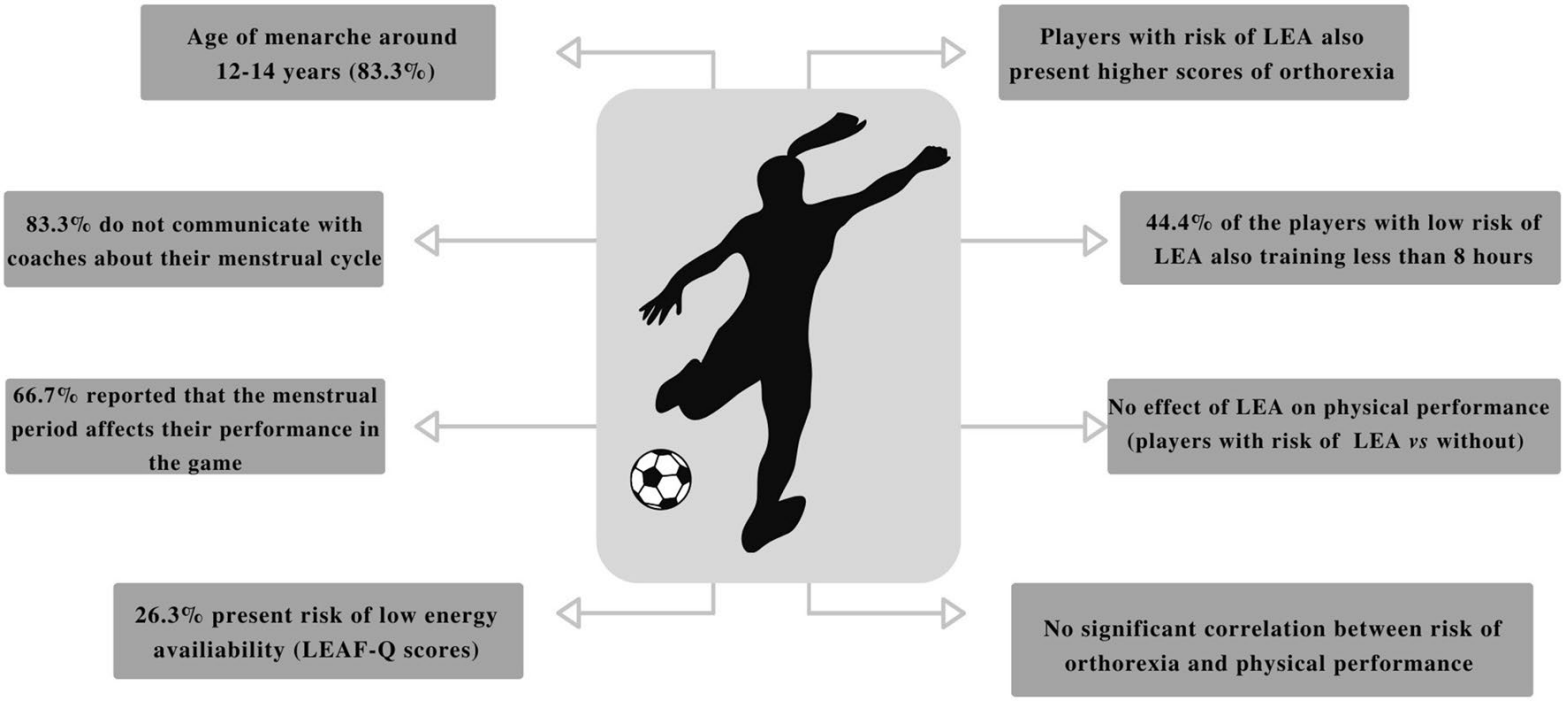
Summary of the main findings.

The main aim of the study was to describe the parameters related to the menstrual cycle status, eating behavior, low energy availability and physical performance in youth female soccer players. The main findings to be highlighted were: (i) most players perceived that the menstrual period affect their performance in the game (66.7%), however, the majority did not communicate with coaches about their menstrual cycle (83.3%); (ii) the prevalence of risk of LEA was 26.3%; (iii) players with risk of LEA also presented higher scores of orthorexia nervosa (ON); (iv) neither the risk of LEA nor high scores of ON seems to impair significantly the players performance pre-season (Fig. 1).

Regarding the menstrual cycle perceptions, a previous study demonstrated that professional athletes perceived that menstruation negatively impacted their performance during the competition^25^. Similarly, most youth players in the study described menstruation as affecting their performance in the game (66.7%). Thus, considering this event may change the players’ performance, it could be important for coaches being aware of it. As reported elsewhere, athletes seem more open to talking about their menstrual cycle with female support staff^25^. Interestingly, in the present study, even with a female head coach and a female strength and conditioning coach, the majority of players reported not talking to them about the menstrual cycle (83.3%), but instead, prefer to talk with their teammates (77.8%). Strategies focused on communication between the coach, team staff and the youth players should be reinforced, in order to advise and support the players, especially in the days of menstruation and during competitions.

As recently described, the prevalence of menstrual irregularities in soccer players can vary from 5 to 20%, and it could be a result of higher training hours, insufficient recovery and poor nutrition^10^. The youth players in the study reported having menarche between 12 and 14 years and seemed to be not affected by the training demands. It can be reinforced by the majority of the players reporting a regular menstrual cycle, however, the study found that 26.3% (n = 5) of players presented a risk of LEA by the LEAF-Q questionnaire. Few studies evaluated the LEA in female soccer players but in the adult population. It has been reported that during the mid-season, the energy availability declined in most players^11^, as well as during heavy training and match day^12^. It reinforces the importance of following youth soccer players throughout the season, in order to monitor the LEA responses and act to reverse or minimize the negative status.

As previously described, the LEA has been associated with poor eating behavior. Indeed, in the current study, the youth players with a risk of LEA also presented high scores of ON, and this outcome was significant and with a clear and large effect (ES = 1.25). Moreover, 44.4% of players that trained less than 8 h presented no risk of LEA, but with no significant association. The ON is a recent eating behavior, and less is known about the prevalence in youth athletes. It was recently reported that higher values of ON can be presented in athletes as well as non-athletes^6^. The present study is the first up to now to describe the values of ON in youth female soccer players. Nonetheless, a previous study evaluate the LEA and eating attitudes in professional soccer athletes and found that negative eating attitudes were observed in athletes with LEA compared to those with higher energy availability^11^.

Despite the significant effect of the LEA and ON, the presence of both seems not to affect the soccer players’ physical performance. A potential impact of LEA on performance could occur due to the impairment of physiological parameters function (e.g., circulatory lactate, reduction of fat-free mass, electrolytes abnormality and dehydration)^26^. To date, this is the first study that describes LEA and physical performance in youth soccer players. It needs to be pointed out that even with no significant differences between the group with risk of LEA and low risk, players with risk presented lower results on the jump and cardiorespiratory tests, with moderate and large effects, respectively, but unclear. A study with female junior swimmers demonstrated that, during 12 weeks of the competitive season, those athletes that presented energy deficit were associated with ovarian suppression, and a decline of 9.8% in sports performance (400-m time trial swim test), compared to their athletes with normal cycling, that presented an improvement of 8.2%^27^. A long-term monitoring of LEA and physical performance in youth soccer players is suggested, in order to identify if a chronic LEA affects physical performance.

As the first study that described and associated the menstrual cycle status, LEA, ON and physical performance, limitations should be highlighted. The evaluation of one team might reflect something other than other youth soccer players. A larger sample could also increase the statistical power. A significant effect could be found between LEA and physical performance if a large sample, considering that the effect on the jump and cardiorespiratory tests on players with a risk of LEA players was strong but not clear. Secondly, the study evaluated the players in only one period, during the pre-season so deep inference is not possible. Monitoring the variables throughout the whole season and also long-term could result in different outcomes on LEA and ON, as well as in association with physical performance.

## Conclusion

In conclusion, the study demonstrated that the majority of youth players from a soccer team presented a normal menstrual cycle, perceiving an effect in performance during the games, but with no communication about it with the coach. Also, 26.3% presented a risk of LEA together with significantly higher values of ON, compared to those with a non-risk of LEA. However, even with the risk of LEA and high values of ON, the physical performance was not significantly affected. Although the study is the first to describe the topic, the current data provides a ‘snapshot’ assessment of youth soccer players and thus may not represent the long-term LEA and ON, therefore, the possibility that players altered their responses throughout the season cannot be overlooked.

## Supplementary Information

Supplementary Information.

## Data Availability

The datasets used and/or analysed during the current study available from the corresponding author on reasonable request.
