# Supplementary material for: Description of the menstrual cycle status, energy availability, eating behavior and physical performance in a youth female soccer team
Source: Sci Rep. 2023 Jul 11;13:11194. doi: 10.1038/s41598-023-37967-4 (PMC10336125; doi:10.1038/s41598-023-37967-4)
Supplement: Supplementary file 1 — Supplementary Information. [file 41598_2023_37967_MOESM1_ESM.docx]

Survey Female Athlete

Dear athlete, this part of the questionnaire is a collaboration with Masaryk University Faculty of Sport Studies in Czech Republic and The UCLan Cyprus, from Sports Science department. It has been ethically approved. The information provided by you in this survey will be used only for research purposes.

Your sincerely,
Ana Carolina Paludo - Postdoctoral Researcher from Faculty of Sports Studies Masaryk University (Czech Republic). carolina.paludo@fsps.muni.cz.

Marta Guimonová – Associate Professor from Faculty of Sports Studies - Masaryk University (Czech Republic). gimunova@fsps.muni.cz.

Koulla Parpa - Assistant Professor from Sport and Exercise Science - UCLan in Cyprus

Descriptive characteristics

Name: ___________________________________________ Age: ___________

At what age are you start to play soccer (years)? :__________

Training information and performance

How many hours do you train soccer per week?

( ) less than 8 hours. ( )8 - 12 hours ( )more than 20 hours

Sex of your coach

( ) male ( ) female

Athletes’ natural cycle: perception and communication

During the menstruation days, do you feel that this period affects your performance during the training?

( )Yes ( ) No

During the menstruation days, do you feel that this period affects your performance during the game?

( )Yes ( ) No

Communication with coach and teammates

Do you talk about your menstrual cycle with your coach?

( )Yes ( ) No

Do you talk about your menstrual cycle with your teammates?

( )Yes ( ) No
